# Heart rate n-variability (HRnV) measures for prediction of mortality in sepsis patients presenting at the emergency department

**DOI:** 10.1371/journal.pone.0249868

**Published:** 2021-08-30

**Authors:** Nan Liu, Marcel Lucas Chee, Mabel Zhi Qi Foo, Jeremy Zhenwen Pong, Dagang Guo, Zhi Xiong Koh, Andrew Fu Wah Ho, Chenglin Niu, Shu-Ling Chong, Marcus Eng Hock Ong

**Affiliations:** 1 Duke-NUS Medical School, National University of Singapore, Singapore, Singapore; 2 Health Services Research Centre, Singapore Health Services, Singapore, Singapore; 3 Institute of Data Science, National University of Singapore, Singapore, Singapore; 4 Faculty of Medicine, Nursing and Health Sciences, Monash University, Melbourne, Australia; 5 Department of Emergency Medicine, Singapore General Hospital, Singapore, Singapore; 6 SingHealth Duke-NUS Emergency Medicine Academic Clinical Programme, Singapore, Singapore; 7 Department of Children’s Emergency, KK Women’s and Children’s Hospital, Singapore, Singapore; Vita Salute University of Milan, ITALY

## Abstract

Sepsis is a potentially life-threatening condition that requires prompt recognition and treatment. Recently, heart rate variability (HRV), a measure of the cardiac autonomic regulation derived from short electrocardiogram tracings, has been found to correlate with sepsis mortality. This paper presents using novel heart rate n-variability (HRnV) measures for sepsis mortality risk prediction and comparing against current mortality prediction scores. This study was a retrospective cohort study on patients presenting to the emergency department of a tertiary hospital in Singapore between September 2014 to April 2017. Patients were included if they were above 21 years old and were suspected of having sepsis by their attending physician. The primary outcome was 30-day in-hospital mortality. Stepwise multivariable logistic regression model was built to predict the outcome, and the results based on 10-fold cross-validation were presented using receiver operating curve analysis. The final predictive model comprised 21 variables, including four vital signs, two HRV parameters, and 15 HRnV parameters. The area under the curve of the model was 0.77 (95% confidence interval 0.70–0.84), outperforming several established clinical scores. The HRnV measures may have the potential to allow for a rapid, objective, and accurate means of patient risk stratification for sepsis severity and mortality. Our exploration of the use of wealthy inherent information obtained from novel HRnV measures could also create a new perspective for data scientists to develop innovative approaches for ECG analysis and risk monitoring.

## Introduction

Sepsis is a potentially life-threatening condition caused by the body’s dysregulated response to infection [1]. Every year, over 50 million people are affected, resulting in over five million deaths worldwide [2]. Prompt recognition and treatment of sepsis has been shown to impact patient outcomes, and guidelines have been developed for its management [3]. There is, however, a need for a rapid method to grade sepsis severity and prognosticate the risk for mortality in septic patients. A quick and accurate triage tool for risk stratification of septic patients presenting at the emergency department (ED) would be invaluable, allowing for greater confidence in clinical decisions, and in guiding management.

Several common disease severity scoring systems that have been utilised in the ED for the prediction of sepsis mortality including the Mortality in ED Sepsis (MEDS) score [4], quick SOFA (qSOFA) [5], and intensive care unit (ICU)-based scores such as the Sequential Organ Failure Assessment (SOFA) score [6], and the well-established Acute Physiology and Chronic Health Evaluation II (APACHE II) score [7]. Although these scoring systems have shown good predictive value, certain limitations have prevented their widespread adoption [8–11]. In recent years, heart rate variability (HRV) measurements derived from electrocardiogram (ECG) tracings have allowed for an alternative and complementary approach to predict sepsis mortality. HRV analysis measures the beat-to-beat variation between each R-R interval on an ECG tracing and reflects the autonomic regulation of the cardiovascular system [12]. Being a non-invasive tool that can be rapidly obtained even from patients who are unable to give a history, HRV has been shown to be dysregulated in sepsis [13] and correlates well with subsequent mortality [14,15]. Indeed, scoring systems that incorporate HRV parameters among its predictors have outperformed traditional clinical indicators and established disease severity scores in predicting sepsis mortality [16–19]. The use of HRV may thus further enhance our ability to stratify for risk of sepsis mortality.

In our previous work [20], we invented novel heart rate n-variability (HRnV) parameters to provide enhanced prognostic information to complement traditional HRV parameters. The proposed HRnV has two measures—HR_*n*_V and HR_*n*_V_*m*_. HR_*n*_V is derived from non-overlapping R-R intervals, while HR_*n*_V_*m*_ is computed from overlapping R-R intervals. For each of the traditional HRV, HR_*n*_V, and HR_*n*_V_*m*_ measures, time domain, frequency domain, and nonlinear analysis will yield its respective set of parameters. An application of the novel HRnV variables demonstrated improved predictive ability for major adverse cardiac events among patients with chest pain presenting at the ED [20].

This paper aims to study the prognostic ability of HRnV measures alongside traditional HRV parameters in predicting the outcomes in septic patients presenting at the ED and comparing the HRnV-based model with existing mortality prediction scores.

## Methods

### Study design and clinical setting

We conducted a retrospective cohort analysis on a convenience sample of patients presenting to Singapore General Hospital (SGH) between September 2014 to April 2017. SGH is the largest hospital in Singapore, with its ED seeing 300 to 500 patients daily. Patients are triaged on presentation at the ED according to a symptom-based Patient Acuity Category Scale (PACS). The PACS system has four levels: PACS 1 patients are critically ill, PACS 2 patients are non-ambulant but stable, PACS 3 patients are ambulant, and PACS 4 patients are non-emergency. This study was approved, and informed consent was waived by the SingHealth Centralized Institutional Review Board (CIRB Ref No.: 2016/2858).

### Study population and eligibility

Patients were included in the study if they were aged 21 years and above, triaged to either PACS 1 or 2 at the ED, suspected to have sepsis as determined by their attending physician, and if they met two or more out of four Systemic Inflammatory Response Syndrome (SIRS) criteria [21,22]. The SIRS criteria (temperature >38°C or <36°C, heart rate >90 beats per minute, respiratory rate >20 breaths per minute, and total white blood cell count >12,000/mm^3^ or <4000/mm^3^) were used despite recent revisions under the Sepsis-3 consensus that recommend for sepsis screening with qSOFA score [1]. This decision was made primarily to allow for comparability with the existing literature. Additionally, subsequent validation studies have disputed the utility of qSOFA over SIRS for sepsis screening in the ED due to its poor sensitivity for septic patients [23–26]. Patients were excluded if their ECGs had non-sinus rhythm, a high noise level (>30% of the entire recording), or if they had a pacemaker or were on mechanical ventilator support.

### Data collection

Five-minute one-lead ECGs were performed on patients who met the inclusion criteria using the ZOLL X Series monitor/defibrillator (ZOLL Medical Corporation, Chelmsford, MA). In addition, patient demographics, vital signs taken at triage, medical history, and laboratory investigations performed in the ED were retrieved from the electronic medical records. We defined the primary outcome as 30-day in-hospital mortality (IHM).

### HRnV measure and analysis

**Fig 1:**
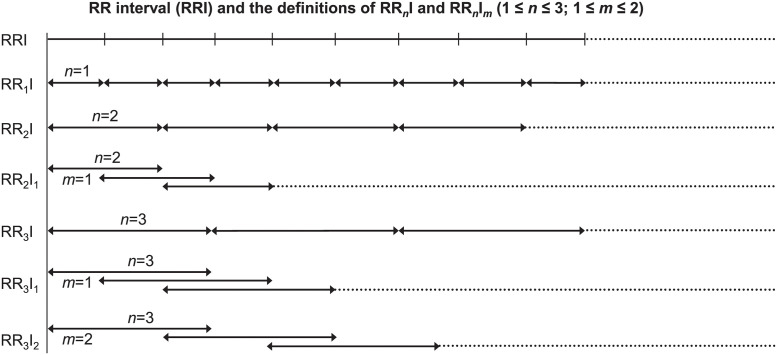
Illustration of the RR intervals (RRIs) and the definitions of RR_*n*_I and RR_*n*_I_*m*_, where 1 ≤ *n* ≤ 3, 1 ≤ *m* ≤ 2. Parameter *m* indicates the non-overlapping portion between two successive RR_*n*_I_*m*_ sequences.

We processed the ECGs and detected QRS complex to convert the original ECG signals into R-R interval (RRI) sequences (i.e., intervals of consecutive R peaks in ECGs). Fig 1 illustrates the definitions of RRI and the derived RR_*n*_I and RR_*n*_I_*m*_ sequences. Conventional HRV analysis evaluates consecutive single RRIs in ECGs. Novel HRnV measures (HR_*n*_V and HR_*n*_V_*m*_) analyse consecutive combined RRIs (RR_*n*_I and RR_*n*_I_*m*_) [20].

To define the HR_*n*_V measure, a new type of RRI called RR_*n*_I is obtained, where *n* is an integer between 1 and *N* and *N* (the number of conventional RRIs combined to form a new RR_*n*_I; for example, RR_2_I is a combination of 2 consecutive RRIs) is much smaller than $\hat{N}$ (total number of RRIs). With newly generated RR_*n*_I sequences, traditional time and frequency domains, and nonlinear analyses [27,28] are applied to calculate HR_*n*_V parameters. In addition to conventional HRV parameters, HR_*n*_V also evaluates two newly created parameters: NN50*n* and pNN50*n*. These two parameters differ from the traditional NN50 and pNN50 parameters in that the threshold is changed from 50 ms to 50×*n* ms in describing the absolute difference between successive RR_*n*_I sequences.

Similarly, HR_*n*_V_*m*_ is a measure derived from RR_*n*_I_*m*_, where *m* is the number determining non-overlapping RRIs for each RR_*n*_I. When *m* = *n*, RR_*n*_I_*m*_ becomes RR_*n*_I as there are no overlapping RRIs, resulting in an upper limit of *N*-1 for *m*. Fig 1 depicts a scenario when *n* = 3 and *m* can be 1 or 2. Utilising all permissible combinations of *n* and *m*, *N*(*N*+1)/2 sets of traditional HRV, novel HR_*n*_V and HR_*n*_V_*m*_ parameters can be generated from a single RRI sequence. Our analysis set the upper limit of *N* as three due to the relatively short duration of collected ECG samples. As a result, one set of HRV parameters, two sets of HR_*n*_V (HR_2_V and HR_3_V) parameters, and three sets of HR_*n*_V_*m*_ (HR_2_V_1_, HR_3_V_1_, and HR_3_V_2_) parameters were calculated. The HRnV-Calc software suite (https://github.com/nliulab/HRnV) was used for calculating the HRV and HRnV parameters, in which the functions from PhysioNet Cardiovascular Signal Toolbox [29] were performed for ECG signal processing.

### Statistical analysis

Categorical variables were compared between patients who did and did not meet the primary outcome (30-day IHM) using χ^2^ test or Fisher’s exact test where appropriate. Continuous variables were checked for normality with the Kolmogorov-Smirnov test. Subsequently, normally distributed variables were presented as mean and standard deviation (SD) and were compared with independent two-tailed *t* test between groups, while non-normally distributed variables were presented as median and interquartile range (IQR; 25^th^ to 75^th^ percentiles) and compared using the Mann-Whitney U test.

Univariable regression analysis was conducted on traditional HRV parameters, novel HRnV parameters and demographic and clinical variables. Each variable was evaluated as an individual predictor of the primary outcome (30-day IHM) using binary logistic regression with odds ratio (OR), 95% confidence interval (CI), and p-value reported. For multivariable regression analysis, we adjusted for age, temperature, systolic blood pressure, heart rate, and Glasgow Coma Scale (GCS) as these variables were either shown to be significant predictors of sepsis mortality in previous literature [15,30–32], or are included in well-established sepsis scoring systems such as the National Early Warning Score (NEWS) [33], Modified Early Warning Score (MEWS) [34], qSOFA, or APACHE II. HRV and HRnV parameters were included in the multivariable analysis if they achieved p<0.2 in the univariable analysis. Included variables were then checked for collinearity using Pearson’s R correlation. For each collinear pair, the variable with the higher p-value on univariate analysis was eliminated until no collinear pairs remained.

The remaining variables were then fed into a backward stepwise multivariable logistic regression model, which used p<0.1 as an endpoint. We took statistical significance at p<0.05. Backward elimination was chosen for our stepwise variable selection because it has the advantage to assess the joint predictive ability of variables, and it removes the least essential variables. However, the eliminated variables cannot re-enter the model [35]. In comparison, all possible subset selection examines every combination of variables, requiring tremendous computing resources yet likely overfitting the model when the number of variables is large [35].

In predictive modelling with the selected variables, we conducted 10-fold cross-validation to avoid overfitting in evaluating models. We split the entire dataset into 10 non-overlapping subsets of equivalent size and then used nine subsets to build a model and validated the model with the remaining one subset. We repeated the above process ten times to ensure that each of the ten subsets could be validated. Subsequently, a receiver operating characteristic (ROC) curve was plotted to assess the predictive ability of the multivariable regression model and compared against other established disease scoring systems on their area under the curve (AUC).

Missing data were addressed by median imputation, in consideration of the low proportion of missing data (<0.3%) for each variable, the nature of variables, and recommendations for missing data in clinical trials [36]. There were three missing observations for which the median value was imputed; one patient had an unknown medical history of cancer, and another patient was missing both initial and worst qSOFA scores.

All statistical analyses were carried out using Python version 3.8.0 (Python Software Foundation, Delaware, USA) using the SciPy library (version 1.3.1). Regression models were built using the StatsModels library (version 0.10.2) and scikit-learn library (version 0.22). All methods were implemented in accordance with relevant guidelines and regulations.

## Results

### Patient recruitment

**Fig 2:**
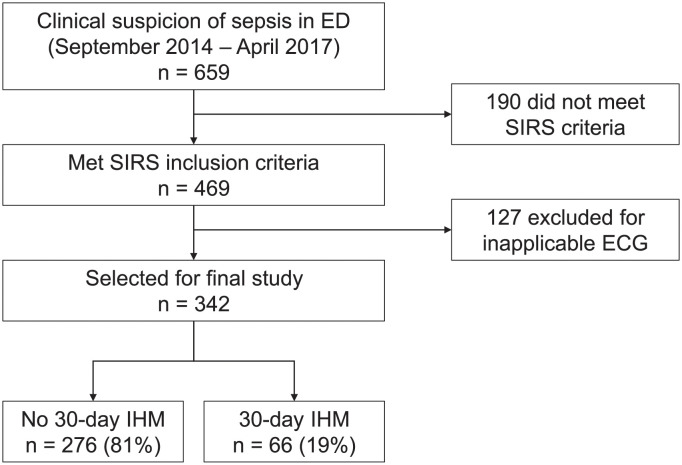
Patient recruitment flowchart. ECG: Electrocardiogram; ED: Emergency department; IHM: In-hospital mortality; SIRS: Systemic inflammatory response syndrome.

Fig 2 presents the patient recruitment flowchart. Of the 659 patients that were initially recruited, 190 patients did not meet the SIRS criteria, and 127 patients had inapplicable ECG readings. Three hundred forty-two patients were included for analysis and classified depending on whether they met the primary outcome of 30-day IHM (n = 66, 19%) or did not meet the primary outcome (n = 276, 81%).

### Baseline characteristics and clinical parameters

Table 1 illustrates baseline characteristics and clinical parameters of patients who met and did not meet with 30-day IHM. Patients who met with 30-day IHM were older and presented with higher respiratory rates but lower temperatures, systolic blood pressures (SBP) and GCS scores, compared to patients who did not meet with 30-day IHM. The worst recorded values of respiratory rate, GCS, and SBP during each patient’s ED stay were also significantly more abnormal in patients that met with 30-day IHM. The difference in disposition from the ED was significant, with a larger proportion of patients who eventually met with 30-day IHM requiring admission to the ICU as compared to patients who did not meet with 30-day IHM (16.7% vs 4.3%, p = 0.001). Additionally, a larger proportion of patients who met with 30-day IHM had a respiratory source of infection (45.5% vs 27.2%, p = 0.006) while a smaller proportion had a source of infection originating from the urinary tract (7.6% vs 25.7%, p = 0.003) when compared to patients who did not meet with 30-day IHM. No significant differences were detected in gender, PACS status, ethnicity, or medical history between both groups.

**Table 1:** Baseline characteristics and clinical parameters.

Variable	No 30-day IHM (n = 276)	30-day IHM (n = 66)	*P*-value
Age, mean (SD)	65.8 (16.1)	73.2 (14.8)	0.001*
Male gender, n (%)	144.0 (52.2)	30.0 (45.5)	0.399
Triaged to high acuity (PACS1), n (%)	254.0 (92.0)	64.0 (97.0)	0.19
SIRS criteria met, n (%)	250.0 (90.6)	58.0 (87.9)	0.667
**Race, n (%)**			0.946
Chinese	203.0 (73.6)	49.0 (74.2)	0.967
Malay	40.0 (14.5)	8.0 (12.1)	0.763
Indian	21.0 (7.6)	6.0 (9.1)	0.883
Other	12.0 (4.3)	3.0 (4.5)	1
**Disposition from ED, n (%)**			0.001*
Intensive care unit	12.0 (4.3)	11.0 (16.7)	0.001*
high-dependency unit	28.0 (10.1)	3.0 (4.5)	0.231
General ward	236.0 (85.5)	52.0 (78.8)	0.247
**Medical history, n (%)**			
Ischemic heart disease	73.0 (26.4)	21.0 (31.8)	0.469
Diabetes	111.0 (40.2)	24.0 (36.4)	0.663
Hypertension	156.0 (56.5)	35.0 (53.0)	0.708
Cancer	79.0 (28.6)	23.0 (34.8)	0.399
Serious infection	117.0 (42.4)	28.0 (42.4)	0.894
**Source of infection, n (%)**			
Respiratory	75.0 (27.2)	30.0 (45.5)	0.006*
Urinary tract	71.0 (25.7)	5.0 (7.6)	0.003*
Gastrointestinal	18.0 (6.5)	4.0 (6.1)	1
Musculoskeletal	11.0 (4.0)	3.0 (4.5)	0.738
Hepatobiliary	20.0 (7.2)	0.0 (0.0)	0.018*
Peritoneum	3.0 (1.1)	2.0 (3.0)	0.248
Skin	3.0 (1.1)	0.0 (0.0)	1
Line	7.0 (2.5)	0.0 (0.0)	0.354
Cardiac	7.0 (2.5)	2.0 (3.0)	0.686
Central nervous system	1.0 (0.4)	0.0 (0.0)	1
Unknown	23.0 (8.3)	12.0 (18.2)	0.032*
No infection	37.0 (13.4)	8.0 (12.1)	0.94
**Vital sign predictors, mean (SD) or median (IQR)**			
Heart rate, bpm	114.2 (23.3)	112.7 (26.0)	0.652
White blood cell count	14.0 (7.7)	13.0 (9.6)	0.36
Diastolic BP, mmHg	63.0 (19.5)	59.7 (17.4)	0.213
Temperature, °C	38.1 (37.1–38.8)	37.2 (36.3–38.0)	<0.001*
Respiratory rate, bpm	19.0 (18.0–21.0)	22.0 (19.0–25.0)	<0.001*
Respiratory rate (worst), bpm	21.0 (19.8–24.0)	26.0 (22.0–30.0)	<0.001*
Systolic BP, mmHg	109.0 (86.0–139.0)	101.0 (78.0–118.5)	0.012*
Systolic BP (worst), mmHg	90.0 (77.0–109.2)	78.0 (63.2–94.8)	<0.001*
GCS (3–15)	13.4 (3.0)	11.7 (4.1)	0.001*
Worst GCS (3–15)	13.4 (3.1)	11.7 (4.1)	0.002*

*p<0.05.

IHM: In-hospital mortality; SD: Standard deviation; IQR: Interquartile range; BP: Blood pressure; GCS: Glasgow Coma Scale; ED: Emergency department.

### HRV and HRnV parameter description and univariable analysis

Table 2 presents the descriptive analysis of HRV and HRnV parameters. In this study, *N* was set as 3 and HR_2_V, HR_2_V_1_, HR_3_V, HR_3_V_1_ and HR_3_V_2_ parameters were calculated. Among time domain parameters such as mean NN and SDNN, HR_*n*_V and HR_*n*_V_*m*_ values are generally directly proportional to *n* and increase when *n* increases. HR_2_V SampEn and HR_3_V SampEn were considerably larger than SampEn parameters of HRV, HR_2_V_1_, HR_3_V_1_, and HR_3_V_2_. This was because of insufficient data points since our ECG recordings were only five minutes long. HR_2_V_1_, HR_3_V_1_ and HR_3_V_2_ did not encounter this limitation as more data points were available from a calculation using overlapping RR_*n*_I_*m*_ sequences [20].

**Table 2:** Descriptive analyses of heart rate variability (HRV) and heart rate n-variability (HRnV) parameters.

	HRV	HR_2_V	HR_2_V_1_	HR_3_V	HR_3_V_1_	HR_3_V_2_
Mean NN (ms)	577.32 (135.86)	1150.61 (270.41)	1154.63 (271.70)	1723.85 (406.01)	1731.93 (407.53)	1725.94 (405.63)
SDNN (ms)	22.72 (20.80)	28.90 (25.18)	34.55 (36.99)	35.99 (33.28)	46.02 (53.18)	37.39 (34.43)
Mean HR (bpm)	109.20 (22.75)	54.71 (11.38)	54.55 (11.37)	36.51 (7.60)	36.36 (7.58)	36.46 (7.58)
SD HR (bpm)	4.28 (3.10)	1.38 (1.14)	1.56 (1.22)	0.75 (0.66)	0.91 (0.74)	0.78 (0.67)
RMSSD (ms)	26.65 (21.38)	24.03 (19.70)	24.94 (20.91)	24.75 (20.61)	24.97 (20.76)	24.17 (19.72)
Skewness	0.02 (3.33)	-0.21 (2.25)	-0.11 (2.61)	-0.16 (2.09)	-0.10 (2.10)	-0.16 (1.97)
Kurtosis	24.80 (62.04)	12.36 (28.30)	16.65 (38.92)	10.03 (22.30)	12.08 (25.82)	10.02 (22.53)
Triangular index	4.47 (3.17)	5.92 (4.16)	6.78 (4.54)	6.75 (4.16)	8.77 (5.94)	7.29 (4.73)
NN50 (count)	44.08 (72.93)	18.34 (34.43)	36.86 (69.62)	12.51 (23.53)	37.31 (69.48)	18.85 (35.29)
pNN50 (%)	7.56 (13.29)	6.43 (12.67)	6.46 (12.79)	6.65 (13.18)	6.60 (12.90)	6.67 (13.17)
NN50*n* (count)	-	1.54 (5.85)	3.33 (12.00)	1.05 (3.66)	3.34 (10.97)	1.50 (5.23)
pNN50*n* (%)	-	0.64 (2.69)	0.69 (2.74)	0.64 (2.37)	0.69 (2.42)	0.62 (2.29)
Total power (ms^2^)	911.66 (2856.13)	1224.37 (2983.21)	3189.41 (10678.24)	1563.63 (3803.04)	6532.50 (23296.45)	2121.76 (5730.04)
VLF power (ms^2^)	285.42 (1302.27)	441.08 (1533.75)	1135.39 (5206.55)	654.27 (2024.13)	2545.77 (11693.83)	976.18 (3417.38)
LF power (ms^2^)	188.81 (612.89)	338.39 (915.03)	706.26 (2355.90)	357.25 (862.83)	1496.80 (5051.87)	605.97 (1723.07)
HF power (ms^2^)	256.53 (719.39)	316.90 (617.51)	631.87 (1365.09)	345.05 (986.15)	892.93 (1977.84)	252.99 (488.08)
LF power norm (nu)	36.81 (18.49)	42.85 (17.77)	42.43 (18.63)	56.43 (17.98)	51.19 (18.96)	61.24 (16.92)
HF power norm (nu)	63.19 (18.49)	57.15 (17.77)	57.57 (18.63)	43.57 (17.98)	48.81 (18.96)	38.76 (16.92)
LF/HF	0.86 (1.11)	1.00 (0.93)	1.11 (1.43)	1.83 (1.75)	1.72 (2.35)	2.31 (2.10)
Poincaré SD1 (ms)	18.63 (14.60)	17.08 (14.23)	17.38 (14.09)	18.13 (16.08)	17.45 (14.19)	17.23 (14.24)
Poincaré SD2 (ms)	24.51 (25.84)	35.89 (32.43)	44.16 (49.92)	44.12 (46.37)	61.00 (73.15)	48.15 (45.59)
Poincaré SD1/SD2 ratio	0.87 (0.38)	0.52 (0.21)	0.46 (0.20)	0.47 (0.22)	0.35 (0.19)	0.42 (0.21)
SampEn	1.35 (0.46)	384.67 (5004.12)	1.41 (0.48)	767.92 (7056.05)	1.38 (0.52)	384.66 (5004.12)
ApEn	1.16 (0.22)	0.93 (0.22)	1.13 (0.22)	0.70 (0.23)	1.10 (0.24)	0.90 (0.23)
DFA, α1	0.57 (0.26)	0.83 (0.28)	0.85 (0.28)	1.04 (0.30)	1.09 (0.28)	1.07 (0.30)
DFA, α2	0.83 (0.28)	0.81 (0.39)	0.90 (0.27)	0.55 (0.70)	0.95 (0.26)	0.87 (0.39)

HRV: Heart rate variability; mean NN: Average of R-R intervals; SDNN: Standard deviation of R-R intervals; RMSSD: Square root of the mean squared differences between R-R intervals; NN50: The number of times that the absolute difference between two successive R-R intervals exceeds 50 ms; pNN50: NN50 divided by the total number of R-R intervals; NN50*n*: The number of times that the absolute difference between two successive RR_*n*_I/RR_*n*_I_*m*_ sequences exceeds 50×*n* ms; pNN50*n*: NN50*n* divided by the total number of RR_*n*_I/RR_*n*_I_*m*_ sequences; VLF: Very low frequency; LF: Low frequency; HF: High frequency; SD: Standard deviation; SampEn: Sample entropy; ApEn: Approximate entropy; DFA: Detrended fluctuation analysis.

Table 3 shows the results of univariable analysis of HRV and HRnV parameters. Of 142 HRV and HRnV parameters, 85 were significantly different between the two outcome groups. Specifically, 14 HRV, 14 HR_2_V, 16 HR_2_V_1_, 11 HR_3_V, 16 HR_3_V_1_, and 14 HR_3_V_2_ parameters were statistically significant. In at least four out of six HRnV measures, RMSSD, kurtosis, NN50, pNN50, NN50*n*, pNN50*n*, HF power, HF power norm, Poincare SD1, and Poincare SD1/SD2 were significantly higher, while LF power norm and DFA α2 were significantly lower in patients who met the primary outcome compared to those who did not. Additionally, VLF power and DFA α1 were not significant in HRV analysis but were statistically significant in several HRnV measures.

**Table 3:** Univariable analysis of HRV and HRnV parameters.

	**HRV**		**HR_2_V**		**HR_3_V**	
	**OR (95% CI)**	**p**	**OR (95% CI)**	**p**	**OR (95% CI)**	**p**
Mean NN	1.000 (0.998 to 1.002)	0.903	1.000 (0.999 to 1.001)	0.964	1.000 (0.999 to 1.001)	0.978
SDNN	1.021 (1.008 to 1.035)	0.002*	1.011 (1.002 to 1.020)	0.021*	1.007 (1.000 to 1.014)	0.065
RMSSD	1.021 (1.009 to 1.032)	<0.001*	1.020 (1.008 to 1.032)	0.001*	1.019 (1.007 to 1.030)	0.002*
Skewness	1.019 (0.943 to 1.102)	0.632	0.982 (0.873 to 1.105)	0.762	1.046 (0.916 to 1.193)	0.508
Kurtosis	1.003 (0.999 to 1.007)	0.12	1.007 (0.999 to 1.015)	0.089	1.010 (0.999 to 1.020)	0.063
Triangular index	1.044 (0.967 to 1.128)	0.271	1.029 (0.970 to 1.091)	0.342	1.014 (0.952 to 1.079)	0.668
NN50	1.004 (1.001 to 1.007)	0.016*	1.007 (1.001 to 1.014)	0.034*	1.010 (1.000 to 1.020)	0.048*
pNN50	1.024 (1.006 to 1.042)	0.008*	1.023 (1.004 to 1.041)	0.016*	1.019 (1.002 to 1.037)	0.032*
NN50*n*	-	-	1.055 (1.013 to 1.099)	0.011*	1.087 (1.023 to 1.155)	0.007*
pNN50*n*	-	-	1.122 (1.025 to 1.228)	0.013*	1.136 (1.034 to 1.248)	0.008*
Total power	1.000 (1.000 to 1.000)	0.007*	1.000 (1.000 to 1.000)	0.015*	1.000 (1.000 to 1.000)	0.056
VLF power	1.000 (1.000 to 1.001)	0.079	1.000 (1.000 to 1.000)	0.058	1.000 (1.000 to 1.000)	0.039*
LF power	1.001 (1.000 to 1.001)	0.004*	1.000 (1.000 to 1.001)	0.021*	1.000 (1.000 to 1.001)	0.018*
HF power	1.001 (1.000 to 1.001)	0.004*	1.001 (1.000 to 1.001)	0.001*	1.000 (1.000 to 1.000)	0.268
LF power norm	0.978 (0.962 to 0.995)	0.009*	0.987 (0.972 to 1.002)	0.095	0.986 (0.972 to 1.001)	0.059
HF power norm	1.022 (1.005 to 1.039)	0.009*	1.013 (0.998 to 1.029)	0.095	1.014 (0.999 to 1.029)	0.059
LF/HF	0.682 (0.457 to 1.017)	0.061	0.856 (0.618 to 1.187)	0.352	0.960 (0.810 to 1.138)	0.637
Poincaré SD1	1.027 (1.010 to 1.044)	0.001*	1.028 (1.011 to 1.045)	0.001*	1.021 (1.007 to 1.037)	0.004*
Poincaré SD2	1.013 (1.002 to 1.024)	0.018*	1.007 (1.000 to 1.014)	0.066	1.003 (0.998 to 1.008)	0.235
Poincaré SD1/SD2	2.346 (1.160 to 4.745)	0.018*	8.160 (2.164 to 30.773)	0.002*	8.285 (2.541 to 27.017)	<0.001*
SampEn	0.965 (0.534 to 1.741)	0.905	1.000 (1.000 to 1.000)	0.31	1.000 (1.000 to 1.000)	0.15
ApEn	0.269 (0.083 to 0.879)	0.03*	0.261 (0.079 to 0.862)	0.028*	0.729 (0.232 to 2.288)	0.588
DFA, α1	0.704 (0.243 to 2.038)	0.518	0.342 (0.123 to 0.955)	0.041*	0.213 (0.077 to 0.590)	0.003*
DFA, α2	0.137 (0.047 to 0.400)	<0.001*	0.352 (0.177 to 0.699)	0.003*	0.631 (0.433 to 0.918)	0.016*
	**HR**_**2**_ **V**_**1**_		**HR**_**3**_ **V**_**1**_		**HR**_**3**_ **V**_**2**_	
	**OR (95% CI)**	**p**	**OR (95% CI)**	**p**	**OR (95% CI)**	**p**
Mean NN	1.000 (0.999 to 1.001)	0.905	1.000 (0.999 to 1.001)	0.906	1.000 (0.999 to 1.001)	0.965
SDNN	1.010 (1.003 to 1.018)	0.008*	1.006 (1.001 to 1.012)	0.018*	1.007 (1.000 to 1.013)	0.053
RMSSD	1.022 (1.011 to 1.034)	<0.001*	1.021 (1.010 to 1.033)	<0.001*	1.019 (1.007 to 1.032)	0.002*
Skewness	0.934 (0.842 to 1.036)	0.198	0.909 (0.799 to 1.032)	0.141	0.954 (0.836 to 1.089)	0.485
Kurtosis	1.007 (1.001 to 1.013)	0.022*	1.010 (1.001 to 1.018)	0.03*	1.009 (0.999 to 1.019)	0.092
Triangular index	1.027 (0.972 to 1.085)	0.34	1.012 (0.969 to 1.057)	0.586	1.009 (0.954 to 1.066)	0.763
NN50	1.004 (1.001 to 1.007)	0.022*	1.004 (1.000 to 1.007)	0.034*	1.007 (1.000 to 1.014)	0.043*
pNN50	1.024 (1.006 to 1.042)	0.009*	1.022 (1.004 to 1.040)	0.017*	1.020 (1.002 to 1.038)	0.027*
NN50*n*	1.026 (1.006 to 1.045)	0.009*	1.028 (1.007 to 1.049)	0.007*	1.064 (1.020 to 1.110)	0.004*
pNN50*n*	1.120 (1.028 to 1.220)	0.01*	1.138 (1.036 to 1.249)	0.007*	1.151 (1.044 to 1.270)	0.005*
Total power	1.000 (1.000 to 1.000)	0.015*	1.000 (1.000 to 1.000)	0.022*	1.000 (1.000 to 1.000)	0.031*
VLF power	1.000 (1.000 to 1.000)	0.08	1.000 (1.000 to 1.000)	0.08	1.000 (1.000 to 1.000)	0.059
LF power	1.000 (1.000 to 1.000)	0.005*	1.000 (1.000 to 1.000)	0.005*	1.000 (1.000 to 1.000)	0.026*
HF power	1.000 (1.000 to 1.000)	0.002*	1.000 (1.000 to 1.000)	0.001*	1.001 (1.000 to 1.001)	0.001*
LF power norm	0.980 (0.965 to 0.996)	0.013*	0.979 (0.965 to 0.994)	0.006*	0.979 (0.964 to 0.995)	0.009*
HF power norm	1.020 (1.004 to 1.037)	0.013*	1.021 (1.006 to 1.037)	0.006*	1.021 (1.005 to 1.038)	0.009*
LF/HF	0.769 (0.570 to 1.037)	0.085	0.883 (0.744 to 1.047)	0.153	0.884 (0.752 to 1.040)	0.137
Poincaré SD1	1.029 (1.012 to 1.047)	0.001*	1.028 (1.011 to 1.045)	0.001*	1.027 (1.010 to 1.044)	0.002*
Poincaré SD2	1.006 (1.000 to 1.011)	0.034*	1.004 (1.000 to 1.007)	0.048*	1.004 (0.999 to 1.009)	0.129
Poincaré SD1/SD2	7.893 (2.085 to 29.879)	0.002*	7.405 (1.867 to 29.365)	0.004*	7.610 (2.222 to 26.064)	0.001*
SampEn	0.830 (0.471 to 1.461)	0.518	0.809 (0.480 to 1.364)	0.427	1.000 (1.000 to 1.000)	0.31
ApEn	0.382 (0.122 to 1.192)	0.097	0.728 (0.244 to 2.170)	0.569	0.544 (0.175 to 1.693)	0.293
DFA, α1	0.480 (0.171 to 1.350)	0.164	0.471 (0.173 to 1.284)	0.141	0.380 (0.146 to 0.990)	0.048*
DFA, α2	0.141 (0.047 to 0.423)	<0.001*	0.146 (0.048 to 0.448)	0.001*	0.384 (0.194 to 0.760)	0.006*

* p< 0.05.

HRV: Heart rate variability; OR: Odds ratio; CI: Confidence interval; mean NN: Average of R-R intervals; SDNN: Standard deviation of R-R intervals; RMSSD: Square root of the mean squared differences between R-R intervals; NN50: The number of times that the absolute difference between two successive R-R intervals exceeds 50 ms; pNN50: NN50 divided by the total number of R-R intervals; NN50*n*: The number of times that the absolute difference between 2 successive RR_*n*_I/RR_*n*_I_*m*_ sequences exceeds 50×*n* ms; pNN50*n*: NN50*n* divided by the total number of RR_*n*_I/RR_*n*_I_*m*_ sequences; VLF: Very low frequency; LF: Low frequency; HF: High frequency; SD: Standard deviation; SampEn: Sample entropy; ApEn: Approximate entropy; DFA: Detrended fluctuation analysis.

Overall, six baseline characteristics (age and vital signs at triage including temperature, respiratory rate, SpO_2_, SBP and GCS), 17 HRV parameters, and 96 HRnV parameters had p<0.2 on univariable analysis. After collinearity assessment, the remaining 87 variables were entered into a stepwise-selection regression model.

### Multivariable analysis and ROC analysis

Table 4 presents the multivariable analysis of variables found to be significantly different on univariable analysis. A total of 21 out of 87 variables were selected through stepwise selection. Of the 21 variables, 16 showed p<0.05. These include vital signs such as respiratory rate (OR = 1.168; 95% CI 1.085–1.257; p<0.001), SBP (OR = 0.978; 95% CI 0.966–0.990; p = 0.001), SpO_2_ (OR = 0.892; 95% CI 0.838–0.950; p = <0.001), and GCS (OR = 0.845; 95% CI 0.769–0.929; p = 0.001), and HRnV measures such as HR_2_V_1_ NN50 (OR = 0.808; 95% CI 0.682–0.958; p = 0.014), HR_2_V pNN50 (OR = 0.290; 95% CI 0.115–0.732; p = 0.009), HR_2_V_1_ pNN50 (OR = 5.700; 95% CI 1.784–18.213; p = 0.003), HR_2_V ApEn (OR = 0.106; 95% CI 0.013–0.877; p = 0.037) and several HR_3_V_1_ and HR_3_V_2_ parameters which demonstrated strong predictive power in assessing the risk for 30-day IHM. The final multivariable predictive model consisted of four vital signs, two traditional HRV parameters, and 15 novel HRnV parameters. Hereafter, we refer to this model as the HRnV model.

**Table 4:** Multivariable analysis of HRV and HRnV parameters on 30-day in-hospital mortality.

Variables	Adjusted Odds Ratio (95% CI)	p-value
Vital signs		
Respiratory rate	1.168 (1.085 to 1.257)	<0.001*
Systolic blood pressure	0.978 (0.966 to 0.990)	0.001*
Glasgow coma scale	0.845 (0.769 to 0.929)	0.001*
SpO_2_	0.892 (0.838 to 0.950)	<0.001*
HRV parameters		
HRV total power	1.000 (1.000 to 1.001)	0.019*
HRV Poincare SD1	0.948 (0.893 to 1.007)	0.081
HRnV parameters		
HR_2_V NN50	1.270 (0.967 to 1.667)	0.085
HR_2_V HF power	1.002 (1.000 to 1.003)	0.076
HR_2_V pNN50	0.290 (0.115 to 0.732)	0.009*
HR_2_V ApEn	0.106 (0.013 to 0.877)	0.037*
HR_2_V_1_ NN50	0.808 (0.682 to 0.958)	0.014*
HR_2_V_1_ pNN50	5.700 (1.784 to 18.213)	0.003*
HR_2_V_1_ LF/HF	0.251 (0.071 to 0.880)	0.031*
HR_3_V pNN50	1.229 (0.988 to 1.528)	0.064
HR_3_V_1_ NN50	1.098 (0.999 to 1.206)	0.053
HR_3_V_1_ LF/HF	2.241 (1.252 to 4.013)	0.007*
HR_3_V_1_ pNN50	0.473 (0.247 to 0.906)	0.024*
HR_3_V_1_ NN50*n*	0.794 (0.664 to 0.950)	0.012*
HR_3_V_1_ HF power norm	1.093 (1.044 to 1.144)	<0.001*
HR_3_V_1_ DFA α1	14.189 (1.009 to 199.510)	0.049*
HR_3_V_2_ NN50*n*	1.713 (1.202 to 2.443)	0.003*

*p<0.05.

HRV: Heart rate variability; CI: Confidence interval; NN50: The number of times that the absolute difference between two successive R-R intervals exceeds 50 ms; pNN50: NN50 divided by the total number of R-R intervals; NN50*n*: The number of times that the absolute difference between 2 successive RR_*n*_I/RR_*n*_I_*m*_ sequences exceeds 50×*n* ms; LF: Low frequency; HF: High frequency; SD: Standard deviation; ApEn: Approximate entropy; DFA: Detrended fluctuation analysis.

**Fig 3:**
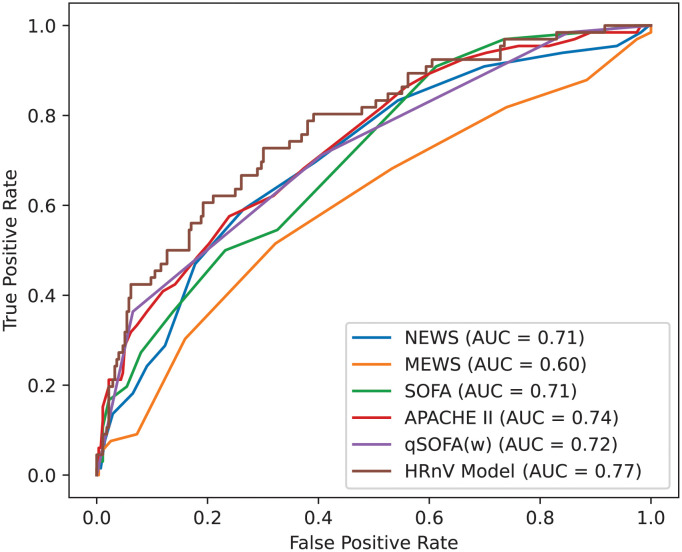
ROC curves for HRnV-based prediction models, alongside other disease severity scoring systems. APACHE II: Acute Physiology and Chronic Health Evaluation II; AUC: Area under the receiver operating characteristic curve; HRnV: Heart rate n-variability; MEWS: Modified Early Warning Score; NEWS: National Early Warning Score; SOFA: Sequential Organ Failure Assessment; qSOFA(w): Worst quick SOFA score.

ROC curves were plotted for assessment of the HRnV model and compared against established disease severity scoring systems to predict 30-day IHM in patients presenting to the ED with sepsis (Fig 3). The AUC of the HRnV model based on 10-fold cross-validation was 0.77 (95% CI: 0.70–0.84), outperforming the AUC of NEWS 0.71 (95% CI: 0.64–0.78), MEWS 0.60 (95% CI: 0.53–0.67), SOFA 0.71 (95% CI: 0.64–0.78), APACHE II 0.74 (95% CI: 0.68–0.80), and the patient’s worst qSOFA value 0.72 (95% CI: 0.65–0.79).

## Discussion

In recent years, there has been a surge in research interest in HRV and its ability to prognosticate for adverse patient outcomes across various disease processes [18,19,28]. To improve the predictive power of HRV, several studies have sought to utilise advanced nonlinear techniques to derive novel HRV parameters [29,30]. Indeed, we previously employed the novel HRnV measures to assess the risk of 30-day major adverse cardiac events in patients presenting to the ED with chest pain [20].

This study evaluated the predictive value of novel HRnV measures (HR_*n*_V and HR_*n*_V_*m*_) in estimating the risk of 30-day IHM in patients presenting to the ED with sepsis. In addition to the 22 traditional HRV parameters, we derived an additional 120 HRnV parameters, 71 of which were found statistically significant in their association with the primary outcome. The newly generated HRnV parameters greatly augment the number of candidate predictors and have demonstrated improved predictive ability for sepsis mortality. Although the physiological meaning and clinical interpretation of some HRnV parameters are yet to discover, the rich inherent information obtained from novel HRnV measures could create a new perspective for data scientists and machine learning researchers to investigate innovative approaches for ECG analysis and risk monitoring.

In HRnV measures, the newly added parameters, NN50*n* and pNN50*n*, were significantly associated with mortality in the univariate analysis. They characterise the number of times that the absolute difference between two successive RR_*n*_I sequences exceeds 50×*n* ms, by assuming the absolute difference may be magnified when the corresponding RR_*n*_I is *n* times longer than RRI [20]. The composite HRnV model derived from multivariable logistic regression achieved the highest AUC on ROC analysis and outperformed other established disease scoring systems such as NEWS, MEWS, SOFA, and APACHE II for the prediction of 30-day IHM in patients presenting to the ED with sepsis.

In addition to demonstrating the superior predictive ability for sepsis mortality, the HRnV model is made even more relevant in its capacity for rapid and objective prognostication where only vital signs and parameters calculated from five-minute ECG tracings are needed. Many established disease severity scores require invasive tests, which need long turnaround time and resources to obtain or include subjective parameters that involve interrater variability while scoring. Among disease severity scores, the MEDS score, explicitly developed for risk stratification of septic patients in the ED, suffers from some of these limitations and its adoption has thus not been widespread. Consequently, MEDS was not included in our comparison. APACHE II and SOFA scores initially designed for use in the intensive care unit (ICU) setting similarly require invasive investigations to calculate its score. In these aspects, the HRnV model which is derived from vital signs taken on ED presentation, and HRV and HRnV parameters calculated from five-minute ECG tracings, can overcome these limitations and provide a rapid, objective, and accurate risk assessment of the septic patient. A triage tool with these characteristics would be invaluable to the physician and can aid in risk stratification, clinical management, patient disposition, and accurate patient classification for administrative or research purposes. Furthermore, our HRnV analysis and modelling modules can be readily integrated into a monitoring device, making the real-time prediction of sepsis severity a feasible task.

### Limitations

First, a majority of the HRnV variables were removed from predictive modelling with traditional logistic regression, which hindered the release of the power of the novel HRnV representation. Moving forward, we endeavour to explore the use of black box or interpretable machine learning algorithms [37–39] for full utilisation of the HRnV parameters. Second, the difficulty in interpreting the HRnV parameters may pose a challenge in their clinical implementation and adoption. However, data scientists and machine learning practitioners may find these parameters valuable in data mining tasks. Third, we were only able to recruit a convenience sample of all suspected sepsis patients at the ED due to resource constraints and the difficulty of continuous ECG measuring in an emergency setting. Moreover, there was a selection bias in patient recruitment as we only included patients from PACS 1 or 2 triage category. To address this issue, we are planning a prospective study to include all ED sepsis patients. Last, sepsis is a seasonal illness that varies throughout the year, but we were unable to examine the impact of seasonality because Singapore’s weather is warm and humid all year round.

## Conclusions

The use of novel HRV measures (HR_*n*_V and HR_*n*_V_*m*_) can provide additional power to predictive models in the risk stratification of patients who present to the ED with sepsis. When included in a model with other clinical variables, the HRnV model outperforms traditional risk stratification scoring systems as shown in our preliminary results. Prospective multi-centre cohort studies would be valuable in validating the effectiveness of the HRnV parameters. The use of HRnV may allow for a rapid, objective, and accurate means of patient risk stratification for sepsis severity and mortality.
